# Multispecialty comparison of point-of-care-ultrasound use, training, and barriers: a national survey of VA medical centers

**DOI:** 10.1186/s13089-024-00398-x

**Published:** 2025-05-21

**Authors:** Dana M. Resop, Brian Bales, Rebecca G. Theophanous, Jessica Koehler, Jeremy S. Boyd, Michael J. Mader, Jason P. Williams, Robert Nathanson, Zahir Basrai, Elizabeth K. Haro, Rahul Khosla, Erin Wetherbee, Harald Sauthoff, Nilam J. Soni, Christopher K. Schott

**Affiliations:** 1 Department of Emergency Medicine, University of Wisconsin, Madison, WI USA; 2 Emergency Department, William S. Middleton Memorial Veterans Hospital, Madison, WI USA; 3 Department of Emergency Medicine, Vanderbilt University Medical Center, Nashville, TN USA; 4 Department of Emergency Medicine, VA TN Valley Healthcare System—Nashville, Nashville, TN USA; 5 Department of Emergency Medicine, Duke University, Durham, NC, USA; 6 Emergency Medicine, Durham VA, Durham, NC USA; 7 Department of Emergency Medicine, University of Michigan Medical School, Ann Arbor, MI USA; 8 Emergency Medicine, VA Ann Arbor, Ann Arbor, MI USA; 9 Research Service, South Texas Veterans Health Care System, San Antonio, TX USA; 10 Medicine Service, Atlanta VA Medical Center, Atlanta, GA USA; 11 Division of Hospital Medicine, Emory School of Medicine, Atlanta, GA USA; 12 Medicine Service, South Texas Veterans Health Care System, San Antonio, TX USA; 13 Division of Hospital Medicine, University of Texas Health San Antonio, San Antonio, TX USA; 14 Emergency Medicine, VA Greater Los Angeles Healthcare System, Los Angeles, CA USA; 15 Department of Emergency Medicine, David Geffen School of Medicine at UCLA, Los Angeles, CA USA; 16 Division of Pulmonary Diseases & Critical Care Medicine, University of Texas Health San Antonio, San Antonio, TX USA; 17 Department of Pulmonary, Critical Care and Sleep Medicine, George Washington University, Washington, DC USA; 18 Pulmonary and Critical Care Medicine, Washington Veterans Affairs Medical Center, Washington, DC USA; 19 Pulmonary Section, Minneapolis Veterans Affairs Health Care System, Minneapolis, MN USA; 20 Division of Pulmonary, Allergy, Critical Care and Sleep Medicine, Department of Medicine, University of Minnesota, Minneapolis, MN USA; 21 Medicine Service, VA NY Harbor Healthcare System, New York, NY USA; 22 Division of Pulmonary, Critical Care, and Sleep Medicine, New York University Grossman School of Medicine, New York, NY USA; 23 Critical Care Service, VA Pittsburgh Health Care Systems, University Drive C, Mail Route: 124-U, Pittsburgh, PA 15240 USA; 24 Departments of Critical Care Medicine and Emergency Medicine, University of Pittsburgh, Pittsburgh, PA USA

**Keywords:** Point of care ultrasound, POCUS, Barriers, Training, Survey, VA, Veterans Affairs

## Abstract

**Background:**

As more specialties have begun to use Point-of-Care Ultrasound (POCUS) in patient care, hospitals and healthcare systems have been investing increasing resources in POCUS infrastructure (training, equipment, and administration). Since each specialty uses different POCUS applications, healthcare systems seek to identify commonalities and differences between specialties to make thoughtful investments in POCUS infrastructure to support each specialty’s use of POCUS while minimizing redundancies. Historically, past studies have focused on POCUS use in individual specialties, primarily emergency medicine and critical care, but comparative studies of different specialties are needed to guide investment in POCUS infrastructure and bolster POCUS implementation across healthcare systems. We conducted a cross-sectional survey of all Veterans Affairs (VA) medical centers in the United States and compared data from 5 different specialties on current usage, training needs, and barriers to POCUS implementation.

**Results:**

Data were collected from facility chiefs of staff (n = 130; 100% response rate) and chiefs of emergency medicine (n = 101; 92% response rate), critical care (n = 93; 83% response rate), hospital medicine (n = 105; 90% response rate), anesthesiology (n = 96; 77% response rate), and surgery (n = 104; 95% response rate). All specialties surveyed reported current POCUS use (surgery 54%, hospital medicine 64%, anesthesiology 83%, emergency medicine 90%, and critical care 93%) but more importantly, a greater desire for training was seen. Procedural POCUS applications were most often used by all specialties, despite decreased procedural POCUS use since 2015 for all specialties except critical care. Diagnostic POCUS use generally increased from 2015 to 2020, although use of specific POCUS applications varied significantly between specialties. Barriers limiting POCUS use included lack of training (53–80%), access to ultrasound equipment (25–57%), and POCUS infrastructure (36–65%).

**Conclusions:**

From 2015 to 2020, POCUS use increased significantly in emergency medicine, critical care, internal medicine, anesthesiology, and surgery, although use of specific applications varied significantly between specialties. Lack of training and POCUS infrastructure were common barriers to POCUS use across specialties. Desire for training exceeded current use for several POCUS applications. These findings can guide implementation and standardization of  POCUS use  in hospitals and healthcare systems.

**Supplementary Information:**

The online version contains supplementary material available at 10.1186/s13089-024-00398-x.

## Background

Point-of-care ultrasound (POCUS) use has expanded significantly over the last two decades since initial implementation of the Focused Assessment with Sonography for Trauma (FAST) exam in emergency departments [1]. Incorporation of POCUS into routine clinical care has been recommended to improve patient-centered outcomes by several medical and surgical specialty organizations [2–18]. Real-time ultrasound guidance for bedside procedures has been shown to reduce procedural complication rates*,* while diagnostic POCUS use has demonstrated decreased time to diagnosis*,* decreased lengths of stay in emergency departments and hospitals, and potentially decreased healthcare costs [19–24]. The importance of POCUS is reflected in several specialty guidelines and integration of POCUS training into medical school, residency, and fellowship curricula. [25–29]

Despite its demonstrated benefits, several barriers to POCUS implementation have been reported with the most common barriers being lack of training and lack of institutional POCUS administration [30–39]. Institutional support of POCUS use, including equipment, training, and infrastructure (image archiving, credentialing pathways, documentation), can improve patient care, quality assurance, and provider communication. The lack of institutional infrastructure has been a rate-limiting barrier to POCUS implementation despite increasing numbers of specialties utilizing POCUS. Hospitals and healthcare systems interested in investing resources in POCUS infrastructure may streamline investments by avoiding redundancies in infrastructure while supporting each specialty’s unique POCUS needs.

Historically, past studies have described POCUS use for a single specialty, but comparative data of POCUS use are needed to better understand the commonalities and differences across specialties. These data will allow healthcare systems to make strategic, deliberate investments in POCUS training, equipment, and infrastructure, including credentialing/privileging policies and image archiving. [34–39]

We conducted a cross-sectional  study of all Veterans Affairs (VA) medical centers in the United States and compared data on current use, training needs, and barriers to POCUS use across 5 acute care specialties: emergency medicine (EM), critical care medicine (CCM), hospital medicine (HM), anesthesiology, and general surgery. Our primary aim was to compare data across these specialties and identify similarities and differences that may be used to guide investment in POCUS infrastructure and facilitate POCUS implementation in healthcare systems.

## Methods

We performed a cross-sectional survey of all VA medical centers between June 2019 and March 2020. A multidisciplinary POCUS workgroup of physicians specializing in EM, CCM, HM, and pulmonary medicine collaborated with the VA’s Healthcare Analysis and Information Group to disseminate a web-based survey systemwide (Verint Systems, Inc.® 2019). The Institutional Review Board of the University of Texas Health San Antonio reviewed and deemed this study to be non-research (Protocol Number: HSC20210630NRR).

The web-based survey included questions on current use, barriers to use, institutional support, equipment, and training needs of POCUS. The survey was deployed in two phases. First, chiefs of staff (n = 130) of VA medical centers nationwide completed 10 questions on facility-level POCUS use, training, competency, and policies between August and October 2019. Next, all specialty chiefs (“chiefs”) representing their specialty’s clinical service at a VA medical center, received a follow-up survey with 18 questions on current specialty-level POCUS use, training needs, workflows, and equipment availability, including specialty-specific questions on core POCUS applications. Individual surveys were sent to specialty chiefs of EM (n = 110), CCM (n = 144), HM (n = 117), anesthesiology (n = 124), and general surgery (n = 109). Data reported by chiefs of intensive care units (ICUs) from sites with multiple ICUs were pooled by facility for comparison with other specialties. The survey period for specialty chiefs started in December 2019 but ended early in March 2020 due to the Covid-19 pandemic.

Data were compared for EM, CCM, HM, anesthesiology, and surgery, which are acute care, hospital-based specialties that are known to use POCUS regularly. In 2015, we conducted a similar cross-sectional survey of all VA medical centers. Instead of querying all specialty chiefs in 2015, the chiefs of staff identified specialties they knew were using POCUS and forwarded the survey to the specialty chiefs. To account for this difference in data collection, subgroup analyses of specialties that answered both the 2015 and 2020 surveys were conducted to assess trends in POCUS use. We compared data from 57 EM groups, 39 ICUs, 24 HM groups, 50 anesthesiology groups, and 54 surgery groups. The survey questions from 2015 and 2020 are available in Appendix 1.

## Results

We received high survey response rates from chiefs of staff (n = 130; response rate 100%) and specialty chiefs of EM (n = 101; response rate 92%), CCM (n = 93, representing 122 ICUs at 93 sites; response rate 83%), HM (n = 105; response rate 90%), anesthesiology (n = 96; response rate 77%), and surgery (n = 104; response rate 95%). The data analyzed came from the specialty chiefs confirming current POCUS use at their sites: EM 89% (n = 90), CCM 93% (n = 113), HM 64% ([n=67] which was confirmed by direct contact with survey respondents), anesthesiology 83% (n = 80), and general surgery 54% (n = 56). Characteristics of hospitals per responses from specialty chiefs are shown in Table 1. Current POCUS use, training, equipment, and infrastructure are shown in Table 2. Current POCUS use from all surveyed specialties is shown in Appendix 1.

**Table 1:** Characteristics of VA facilities with responses from specialty chiefs in 2020

Characteristics	Emerg Med	Critical Care^1^	Hosp Med	Anesthesia	Surgery
	(n = 101)	(n = 93)	(n = 105)	(n = 96)	(n = 104)
**Number of Medicine Beds**					
< 75	71 (70%)	63 (68%)	78 (74%)	67 (70%)	76 (73%)
**Number of Surgery Beds**					
< 25	56 (55%)	49 (53%)	63 (60%)	58 (60%)	62 (60%)
**Number of Operating Rooms**					
0–5	31 (31%)	24 (26%)	38 (36%)	32 (33%)	36 (35%)
06-10	55 (54%)	53 (57%)	51 (49%)	49 (51%)	53 (51%)
11-20	15 (15%)	16 (17%)	16 (15%)	15 (16%)	15 (14%)
**Annual ED Patient Census**					
< 10 K	12 (12%)	9 (10%)	21 (20%)	16 (17%)	19 (18%)
10 K—< 20 K	39 (39%)	34 (37%)	36 (34%)	34 (35%)	37 (36%)
20 K—< 30 K	37 (37%)	37 (40%)	35 (33%)	33 (34%)	35 (34%)
30 K +	13 (13%)	13 (14%)	13 (12%)	13 (14%)	13 (12%)
**VHA ICU Level**					
1	49 (49%)	49 (53%)	50 (48%)	42 (44%)	47 (45%)
2	22 (22%)	18 (19%)	15 (14%)	20 (21%)	20 (19%)
3	25 (25%)	22 (24%)	23 (22%)	20 (21%)	22 (21%)
4	4 ( 4%)	4 ( 4%)	6 ( 6%)	6 ( 6%)	6 ( 6%)
No ICU	1 ( 1%)	0 ( 0%)	11 (10%)	8 ( 8%)	9 ( 9%)
**Facility Complexity Level**^2^					
High	79 (78%)	77 (83%)	73 (70%)	70 (73%)	76 (73%)
**Region**					
Northeast	16 (16%)	16 (17%)	17 (16%)	14 (15%)	18 (17%)
Midwest	26 (26%)	23 (25%)	29 (28%)	24 (25%)	27 (26%)
South	38 (38%)	37 (40%)	38 (36%)	39 (41%)	39 (38%)
West	21 (21%)	17 (18%)	21 (20%)	19 (20%)	20 (19%)
**Location**					
Urban	95 (94%)	87 (94%)	94 (90%)	91 (95%)	97 (93%)

^1^Demographic characteristics refer to the entire VA facility. Some facilities have > 1 intensive care unit that responded, but each facility is counted only once in this table. The 122 intensive care units are represented by 93 VA facilities in this table

^2^ High complexity facilities have high levels of patient volume, patient risk, specialists, teaching, and research. Low complexity facilities have medium to low levels of patient volume and patient risk, and some to little teaching or research

**Table 2:** Current POCUS Use, Training, Equipment, and Administration per Specialty

	**Emerg Med**	**Critical Care**^**1**^	**Hosp Med**	**Anesthesia**	**Surgery**
	(n = 101)	(n = 122)	(n = 105)	(n = 96)	(n = 104)
**Current Use**					
≥ 1 Provider uses POCUS in Group	90 (89%)	113 (93%)	67 (64%)	80 (83%)	56 (54%)
% Physicians using POCUS Weighted Mean (s.d.)	45% (35%)	62% (34%)	42% (38%)	77% (35%)	23% (29%)
**No. Attending Physicians in Service**^2^					
Median (IQR)	14 (10 — 21)	9 (5 – 12)	15 (10 – 23)	8 (4 – 11)	10 (6 – 20)
Mean (s.d.)	18.1 (15.1)	12.8 (21.4)	24.3 (34.3)	8.7 (6.2)	16.8 (18.0)
**Board Certified in Specialty**^3^					
Median (IQR)	6 (2 – 14)	5 (2 – 9)	10 (6 – 15)	Not reported	Not reported
Mean (s.d.)	8.6 (8.1)	5.7 (5.0)	11.2 (6.5)		
**Training**					
Desire for POCUS Training	90 (89%)	97 (80%)	84 (80%)	71 (74%)	50 (48%)
Process to Obtain Training	36 (36%)	45 (37%)	36 (34%)	31 (32%)	20 (19%)
% Physicians with POCUS Training^4^					
Via* CME*					
None	16 (17%)	27 (22%)	49 (48%)	22 (24%)	60 (64%)
Some (≤ 50%)	64 (67%)	63 (52%)	47 (46%)	39 (58%)	26 (28%)
Most (> 50%)	15 (16%)	32 (26%)	6 (6%)	32 (34%)	8 (8%)
Via* Residency /Fellowship*					
None	21 (21%)	26 (21%)	50 (48%)	23 (24%)	51 (54%)
Some (≤ 50%)	52 (52%)	61 (50%)	45 (44%)	33 (35%)	25 (26%)
Most (> 50%)	27 (27%)	35 (29%)	8 (8%)	39 (41%)	19 (20%)
**Rotating Residents/Fellows**	55 (54%)	97 (80%)	57 (54%)	51 (53%)	43 (41%)
**Train Residents/Fellows in POCUS**	29 (29%)	69 (57%)	34 (32%)	46 (48%)	22 (21%)
**Equipment**					
Ultrasound Device Availability					
# Handhelds: Facility-owned	8	39	76	2	25
# Handhelds: Provider-owned	2	13	8	2	6
# Non-shared: Laptop or cart machine	119	174	91	229	94
# Groups sharing ultrasound machine	12	56	31	28	39
**Infrastructure**					
POCUS images saved	4 (4%)	17 (14%)	5 (5%)	14 (15%)	5 (5%)
Specialty privileging policy	7 (7%)	3 (2%)	4 (4%)	6 (6%)	6 (6%)
Quality assurance process	9 (9%)	11 (9%)	5 (5%)	17 (18%)	4 (4%)

^1^A total of 122 intensive care units responded and represent 93 VA facilities. Some facilities have > 1 intensive care unit that responded

^2^Number of attending physicians as reported by specialty chiefs. Note that some may have reported full-time equivalents while others reported each part-timer as one physician. Number of attending physicians was estimated for sites that reported no use of POCUS

^3^Number of board-certified physicians in the specialty, as reported by specialty chief, for HM, the number of hospitalists, regardless of board certification

^4^Some specialty chiefs reported “I don’t know”; those facilities were left out when calculating category percentages

Abbreviations: POCUS, point-of-care ultrasound; Med, medicine; Hosp, hospital; CME, continuing medical education

### Current POCUS use and desire for training

We focus on current POCUS use in five acute care specialties across the VA (EM, CCM, HM, anesthesiology, and surgery). However, among all specialties surveyed (n=32), eleven total inpatient and outpatient specialties reported POCUS use at > 50% of surveyed VA facilities. Of interest, ***all*** specialties surveyed had at least one site reporting POCUS use somewhere within the VA medical system (Appendix 1), and ***all*** sites reported desire for more POCUS training.

**Fig. 1:**
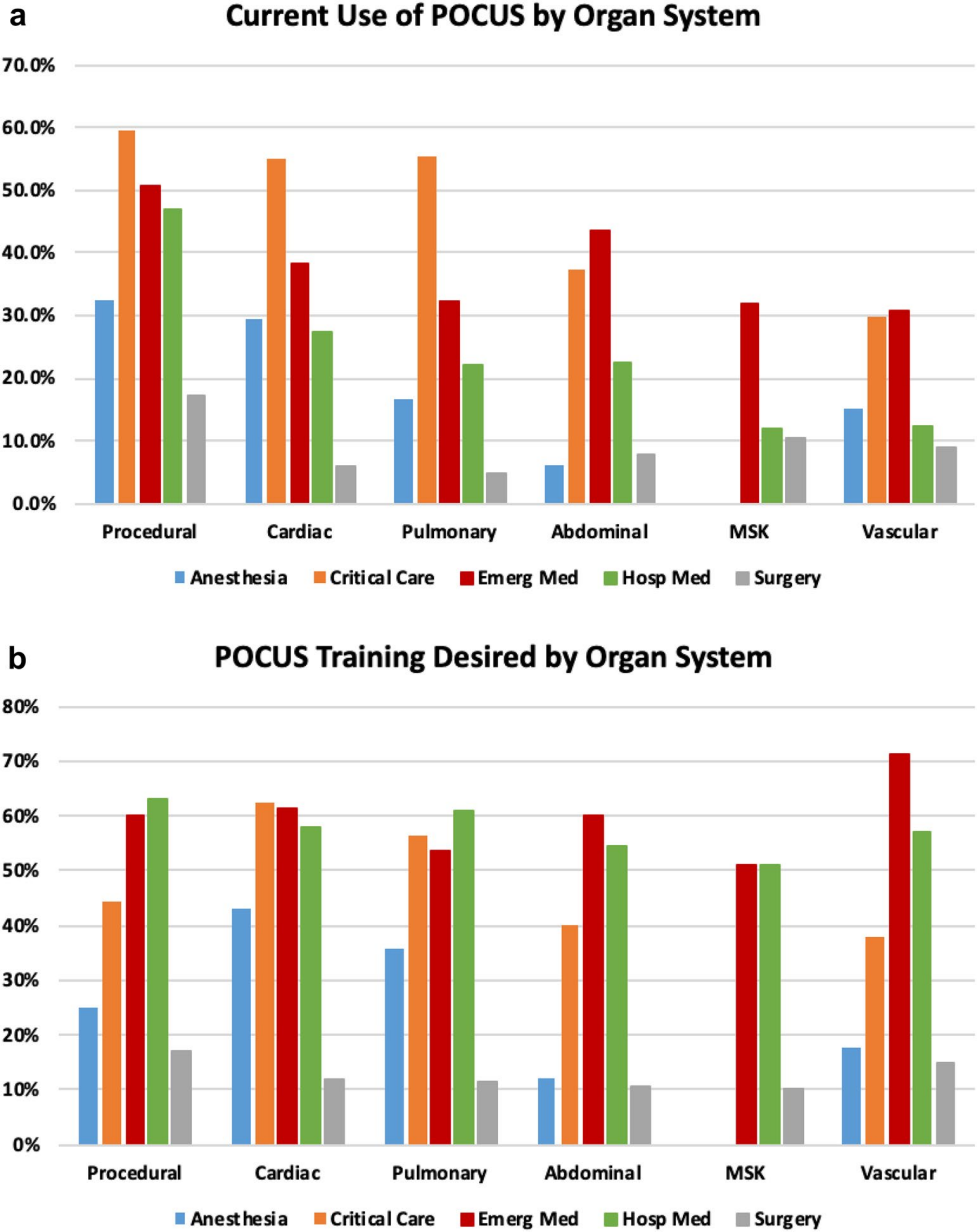
**a** Current POCUS Use by Organ System in Different Specialties. **b**: Desire for POCUS Training by Organ System in Different Specialties. POCUS, Point-of-care Ultrasound; MSK, musculoskeletal; Emerg Med, emergency medicine; Hosp Med, hospital medicine. POCUS applications reporting < 10% use in a specialty were not included in figure

All specialties surveyed reported current POCUS use, defined as ≥ 1 physician in their specialty group using POCUS: 89% in EM, 93% in CCM, 64% in HM, 83% in anesthesiology, and 54% in surgery (Table 2). Figure 1a shows the current POCUS use by organ system for each specialty. Procedural POCUS was the most common application used overall by all specialties. Diagnostic POCUS use varied considerably across specialties. The most common diagnostic POCUS applications used were abdominal for EM; pulmonary for CCM; cardiac for HM and anesthesiology; and musculoskeletal for surgery. Non-surgical specialties utilized diagnostic cardiac POCUS as one of their top 3 applications.

While POCUS use is common across specialties, a high percentage of specialty groups desire POCUS training. Each specialty's desire for POCUS training by organ systems is shown in Fig. 1b. Both anesthesiology (43%) and CCM (63%) groups highly desired more cardiac POCUS training. HM (63%) and surgery (17%) both desired more procedural POCUS training. EM desired more diagnostic vascular (71%) and cardiac (61%) training, followed by procedural POCUS training (60%).

Relatively few (19%–37%) specialty groups had a process to obtain POCUS training (Table 2). EM and CCM had the highest percentage of physicians that obtained their training during residency or fellowship. About half of the specialties surveyed reported training residents and fellows, ranging from 41% in anesthesiology to 80% in CCM. Similarly, POCUS training provided to residents and fellows ranged from 21 to 57% among the specialties with the highest percentage being in CCM (57%).

### Changes from 2015 to 2020

**Fig. 2:**
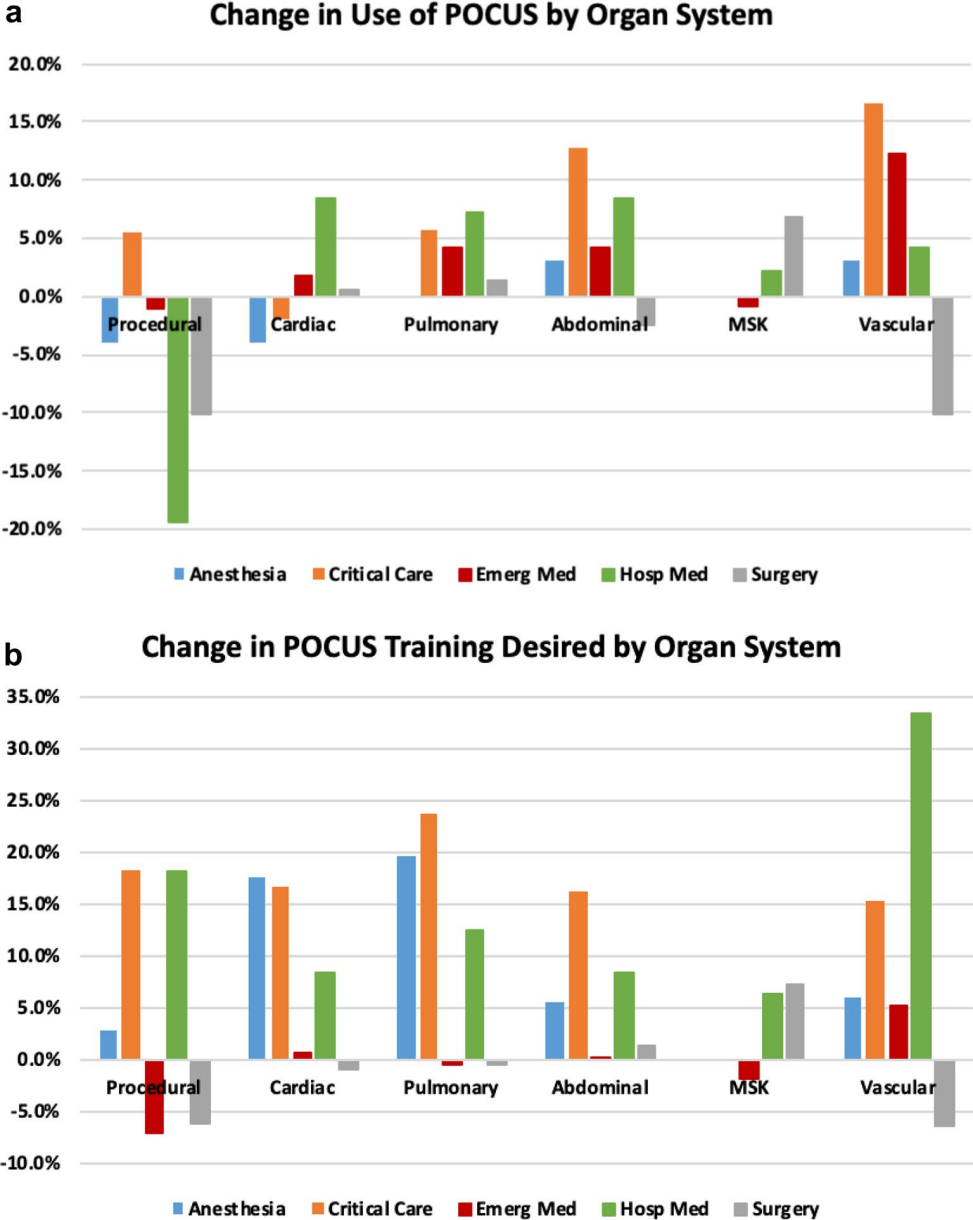
**a** Changes in POCUS Use per Specialty Chiefs from 2015 to 2020. **b** Change in Desire for POCUS Training per Specialty Chiefs from 2015 to 2020. POCUS, Point-of-care Ultrasound; MSK, musculoskeletal; Med, medicine; Hosp Med, hospital medicine

Data on current use and training desired per specialty were compared between 2015 and 2020 (Table 3 and Fig. 2). Diagnostic POCUS use showed an overall increase for most applications across specialties with the greatest increase in vascular POCUS use in EM, CCM, and anesthesiology. Current procedural POCUS use decreased for all specialties, except CCM where it increased in use. Despite the decrease in procedural POCUS use, all specialties, except for surgery and EM, had increased desire for procedural POCUS training.

**Table 3:** Change in POCUS Use and Desire for Training from 2015 to 2020

		Emergency Med (N = 57)		Critical Care (N = 39)		Hospital Med (N = 24)		Anesthesiology (N = 50)		Surgery (N = 54)	
**Current Use of POCUS** (% change)	Procedural	50.7%	(− 1.1%)	59.5%	(5.5%)	47.0%	(− 19.4%)	32.6%	(− 4.0%)	17.0%	(− 10.1%)
	Cardiac	38.3%	(1.8%)	54.9%	(− 1.9%)	27.3%	(8.3%)	29.5%	(− 4.0%)	6.0%	(0.5%)
	Pulmonary	32.4%	(4.1%)	55.5%	(5.8%)	21.9%	(7.3%)	16.8%	(0.0%)	4.8%	(1.4%)
	Abdominal	43.6%	(4.1%)	37.2%	(12.8%)	22.5%	(8.3%)	6.2%	(3.0%)	7.7%	(− 2.4%)
	MSK	31.9%	(− 0.9%)	13.1%	(7.1%)	11.9%	(2.1%)	3.1%	(2.7%)	10.6%	(6.9%)
	Vascular	30.7%	(12.3%)	29.9%	(16.7%)	12.4%	(4.2%)	15.0%	(3.0%)	8.9%	(− 10.2%)
**Desire for Training** (% change)	Procedural	60.0%	(− 7.0%)	44.4%	(18.3%)	63.2%	(18.1%)	25.0%	(2.9%)	16.9%	(− 6.1%)
	Cardiac	61.4%	(0.6%)	62.5%	(16.7%)	58.1%	(8.3%)	43.3%	(17.5%)	12.0%	(− 0.9%)
	Pulmonary	53.8%	(− 0.6%)	56.4%	(23.7%)	61.0%	(12.5%)	35.8%	(19.5%)	11.3%	(− 0.5%)
	Abdominal	60.0%	(0.3%)	40.2%	(16.2%)	54.6%	(8.3%)	12.2%	(5.5%)	10.7%	(1.3%)
	MSK	51.0%	(− 1.8%)	17.4%	(− 0.6%)	51.0%	(6.3%)	4.9%	(2.0%)	10.1%	(7.1%)
	Vascular	71.3%	(5.3%)	37.7%	(15.4%)	57.1%	(33.3%)	17.5%	(6.0%)	14.8%	(− 6.5%)

POCUS, point-of-care ultrasound; Med, medicine; MSK, musculoskeletal

Changes in desire for diagnostic POCUS training also varied by specialty. Desire for diagnostic vascular POCUS training had the highest increase in EM and HM, whereas pulmonary POCUS training was most desired by anesthesiology and CCM. Among surgery groups, the greatest change in desire for training was musculoskeletal POCUS. Overall, the greatest desire for training was reported by EM groups, even though relatively small changes were seen from 2015 to 2020.

### POCUS equipment & infrastructure

At least one dedicated laptop or cart-based ultrasound machine was available to most specialties, specifically 94% of EM, 83% of CCM, 70% of HM, 95% of anesthesiology, and 64% of surgery groups. Handhelds were more commonly available in HM (≥ 1 handheld in 36% of HM groups) compared to EM (7%), CCM (19%), anesthesiology (4%), and surgery (13%). Most specialty groups did not have POCUS image archiving, specialty-specific POCUS credentialing/privileging policies, or quality assurance processes (Table 2).

### Barriers

The barriers to POCUS use were similar across specialties (Table 4). A lack of trained clinicians was the most common barrier among all specialties except anesthesiology, where it was the second most common barrier. Training-related barriers, including lack of funding and opportunities for training, were frequently reported across all specialties. All specialties reported at least 1 barrier related to infrastructure (36–65%) or equipment (25–57%).

**Table 4:** Barriers to POCUS Use per Specialty Chiefs

	**Anesthesia**	**Critical Care**^**1**^	**Emergency Med**	**Hosp Med**	**Surgery**
**Barrier**	(n = 96)	(n = 122)	(n = 101)	(n = 105)	(n = 104)
**TRAINING**					
Lack of Trained Providers	32 (33%)	59 (48%)	72 (71%)	91 (87%)	55 (53%)
Lack of Funding for Training	34 (35%)	55 (45%)	51 (50%)	57 (54%)	30 (29%)
Lack of Training Opportunities	27 (28%)	45 (37%)	49 (49%)	56 (53%)	30 (29%)
Lack of Funding for Travel	22 (23%)	40 (33%)	37 (37%)	28 (27%)	17 (16%)
**At least 1 TRAINING Barrier**	**51 (53%)**	**82 (67%)**	**85 (84%)**	**93 (89%)**	**62 (60%)**
**INFRASTRUCTURE**					
Lack of Image Archiving	14 (15%)	42 (34%)	35 (35%)	34 (32%)	13 (13%)
No Clinician Champion	6 ( 6%)	33 (27%)	33 (33%)	35 (33%)	32 (31%)
Lack of Funding for Support Staff	20 (21%)	32 (26%)	28 (28%)	30 (29%)	20 (19%)
Lack of Standard Reporting Form	12 (13%)	25 (20%)	22 (22%)	26 (25%)	6 (25%)
Lack of Funding for Simulation Space	13 (14%)	25 (20%)	17 (17%)	11 (10%)	8 ( 8%)
Lack of Privileging Criteria	4 ( 4%)	22 (18%)	17 (17%)	20 (19%)	11 (11%)
Lack of Facility Leadership Support	5 ( 5%)	16 (13%)	14 (14%)	12 (11%)	8 ( 8%)
At least 1 **INFRASTRUCTURE** barrier	**35 (36%)**	**72 (59%)**	**66 (65%)**	**66 (63%)**	**48 (46%)**
**EQUIPMENT**					
Lack of ultrasound equipment	17 (18%)	36 (30%)	19 (19%)	57 (54%)	43 (41%)
Lack of funding for US equipment	18 (19%)	25 (20%)	18 (18%)	29 (28%)	27 (26%)
At least 1 **EQUIPMENT** barrier	**24 (25%)**	**43 (35%)**	**29 (29%)**	**60 (57%)**	**46 (44%)**
**OTHER**					
No barriers identified	31 (32%)	21 (17%)	3 ( 3%)	6 ( 6%)	18 (17%)
No perceived benefit	11 (11%)	10 ( 8%)	8 ( 8%)	11 (10%)	19 (18%)

Bold numbers/percentages represent reporting of any one of the barriers listed in the subsection above

^1^ A total of 122 intensive care units responded and represent 93 VA facilities. Some facilities have > 1 intensive care unit that responded

## Discussion

We have conducted the largest comparative study of current use, training needs, and barriers to POCUS use in EM, CCM, HM, anesthesiology, and surgery in the VA healthcare system. Our findings can provide guidance to hospitals and healthcare systems seeking to implement and standardize POCUS use across specialties in large healthcare systems.

Currently, POCUS use appears to be universal across all specialties surveyed. However, our data identified important similarities and differences in POCUS use by specialty that should be considered when developing systemwide initiatives to implement POCUS use. Identifying areas of overlap in POCUS use between specialties and pooling learners and instructors from varied specialties for training courses can facilitate implementation of standard POCUS practices. For instance, a majority of specialties utilize POCUS for procedural guidance, and all EM, CCM, HM, anesthesiology, and surgery groups currently use ultrasound for vascular access. Hence, standardization of procedural POCUS training is possible by training clinicians through focused workshops that teach use of the same, consensus-based techniques and documentation templates for specific procedures. In contrast, some POCUS training is specialty specific. For instance, musculoskeletal ultrasound was a relatively common diagnostic application among surgery and rheumatology groups. However, surgery groups use musculoskeletal ultrasound primarily for diagnosis and management of subcutaneous abscesses, whereas rheumatology groups use musculoskeletal ultrasound frequently to evaluate joints, synovia, and tendons. Thus, efforts to standardize POCUS use of applications with varied use across specialties, such as musculoskeletal ultrasound, should focus on standardization of practice among clinicians within an individual specialty, alongside standardization of specific applications performed by multiple specialties, such as knee and shoulder aspiration/injection among musculoskeletal applications.

Although POCUS use varies by specialty, barriers to POCUS use are relatively consistent across specialties, and it is plausible that addressing the same barriers affecting multiple specialties could facilitate implementation across healthcare systems. The VA, like most healthcare systems, does not have system- or facility-level leadership for POCUS, resulting in heterogeneous equipment acquisition, training, and clinical use. Consistent with this, all specialties identified lack of training, including lack of funding and opportunities for training, followed by lack of infrastructure, including equipment and image management, as top barriers to POCUS use.

Lack of training was the most prevalent barrier with > 50% of chiefs reporting a training-related barrier. Many community and academic healthcare settings have also reported lack of training as a top barrier to POCUS use [33,40]. To promote safe, evidence-based POCUS use, large healthcare systems, such as the VA, can support training physicians in-practice by creating POCUS training programs, including educational support in clinical environments for skill development and maintenance [41]. This approach can provide support for multiple specialties with a few caveats based on our study’s findings. Most important, training curricula should be tailored to each specialty’s needs because current use and desire for training vary by specialty and appear to evolve as seen in our data from 2015 to 2020. Therefore, we believe developing POCUS educational initiatives must involve physicians who actively provide patient care using POCUS within the target specialty. Lack of training among physicians in-practice will not simply improve over time without a deliberate investment in training. For instance, in emergency medicine, a specialty for which a strong justification can be made for POCUS training, the desire for POCUS training did not change significantly from 2015 to 2020 possibly due to absence of systemwide initiatives or pathways for POCUS training. A POCUS training course piloted in the VA healthcare system was effective for acquiring and retaining POCUS knowledge and skills and may serve as a model for future training initiatives [42]. Lastly, specialties that have incorporated POCUS training into Accreditation Council for Graduate Medical Education (ACGME) requirements, including EM, CCM, and anesthesiology, had greater POCUS usage among physicians in-practice [43]. Thus, incorporation of POCUS training into ACGME requirements may be a more sustainable and self-perpetuating approach to implementing routine POCUS use within a specialty.

Lack of POCUS administrative infrastructure, including image archiving, documentation, clinician champions, and policies, were also common barriers identified. Our findings may help hospitals and healthcare systems streamline efforts and costs to address these barriers. By archiving POCUS images, clinicians and healthcare systems benefit from capturing additional clinical revenue, improving clinician-to-clinician and clinician-to-patient communication, streamlining diagnostic workups, reducing redundant diagnostic imaging, and potentially reducing medicolegal risks. Most important, a POCUS image archival allows clinicians to perform quality assurance reviews, and healthcare systems can capture workload to justify expenditures to maintain a POCUS program [44]. A multispecialty collaboration can develop standard workflows for image archiving and documentation which can support each specialty’s use of POCUS. [45–47]

Historically, access to ultrasound equipment was the main barrier to POCUS use with 66% of EM groups reporting no access and 81% reporting shared access to an ultrasound machine in 2003 [40,48]. With increasing availability of high-quality, low-cost portable ultrasound machines, particularly handheld devices, access to ultrasound equipment has become less of a barrier across specialties. HM had a unique predominance of handheld ultrasound devices (45%), which may be explained by hospitalists’ need for small, portable ultrasound devices that can be carried easily to patients located on different hospital floors. Despite the increasing availability of ultrasound equipment, funding for clinician and support staff time to evaluate, procure, and maintain ultrasound equipment is underappreciated and remains an important equipment-related barrier.

We recognize our study has limitations. By partnering with a national professional surveying group, we had high response rates from chiefs of staff (100%) and specialty chiefs (77–95%). However, because the data were self-reported and individual physicians could not be surveyed, the data may not accurately reflect actual clinical practice. Also, our data focused on POCUS use and does not elucidate changes in patient care practices from 2015 to 2020. Although we identified a decrease in procedural POCUS use, our data cannot determine if fewer procedures are being performed by clinicians, or fewer clinicians are utilizing POCUS to guide performance of procedures. Lastly, our data and findings are limited to the VA healthcare system which may limit generalizability to other healthcare systems. However, VA medical centers represent a diverse group of community and academic teaching hospitals that are often staffed by physicians that practice at both VA and non‐VA facilities. Additionally, our data mirror findings from other publications on use and barriers of POCUS in EM, HM, CCM, anesthesiology, and surgery. [49–54]

Future studies shall focus on systematic approaches to address barriers to POCUS use identified by different specialties, including clinician training and ongoing education; skill and knowledge certification; credentialing and privileging practices; establishment of standardized workflows for POCUS documentation, image archiving, and quality assurance; and equipment management. Our study revealed important facility- and specialty-level barriers to POCUS use, and local and national leaders shall address these barriers in an effort to standardize POCUS use systemwide.

## Conclusion

From 2015 to 2020, POCUS use and desire for training have significantly increased in multiple specialties, including EM, CCM, HM, anesthesiology, and surgery, in the VA healthcare system. The most common barriers to POCUS use were lack of training and program infrastructure. Our findings can guide hospitals and healthcare systems seeking to design interventions to implement and standardize POCUS use across multiple specialties in different clinical settings.

## Supplementary Information

Additional file 1.

## Data Availability

2020 Point of Care Ultrasound Survey Data was collected in collaboration with the Health Analysis and Information Group, Department of Veterans Affairs, Milwaukee, Wisconsin, USA. Contacts: Brandy Drum, MBA [17] (brandy.drum@va.gov), Edward O’Brien^17^ (edward.obrien3@va.gov). If needed, the original raw data can be provided.

## Abbreviations

VA: Veterans affairs
POCUS: Point-of-care ultrasound
CCM: Critical care medicine
HM: Hospital medicine
EM: Emergency medicine
ED: Emergency department
ICU: Intensive care unit
MSK: Musculoskeletal
