# Supplementary material for: Multispecialty comparison of point-of-care-ultrasound use, training, and barriers: a national survey of VA medical centers
Source: Ultrasound J. 2025 May 21;17:25. doi: 10.1186/s13089-024-00398-x (PMC12095105; doi:10.1186/s13089-024-00398-x)
Supplement: Supplementary file 1 — Additional file 1. [file 13089_2024_398_MOESM1_ESM.docx]

**Appendix 1: Current POCUS Use in All Specialties**


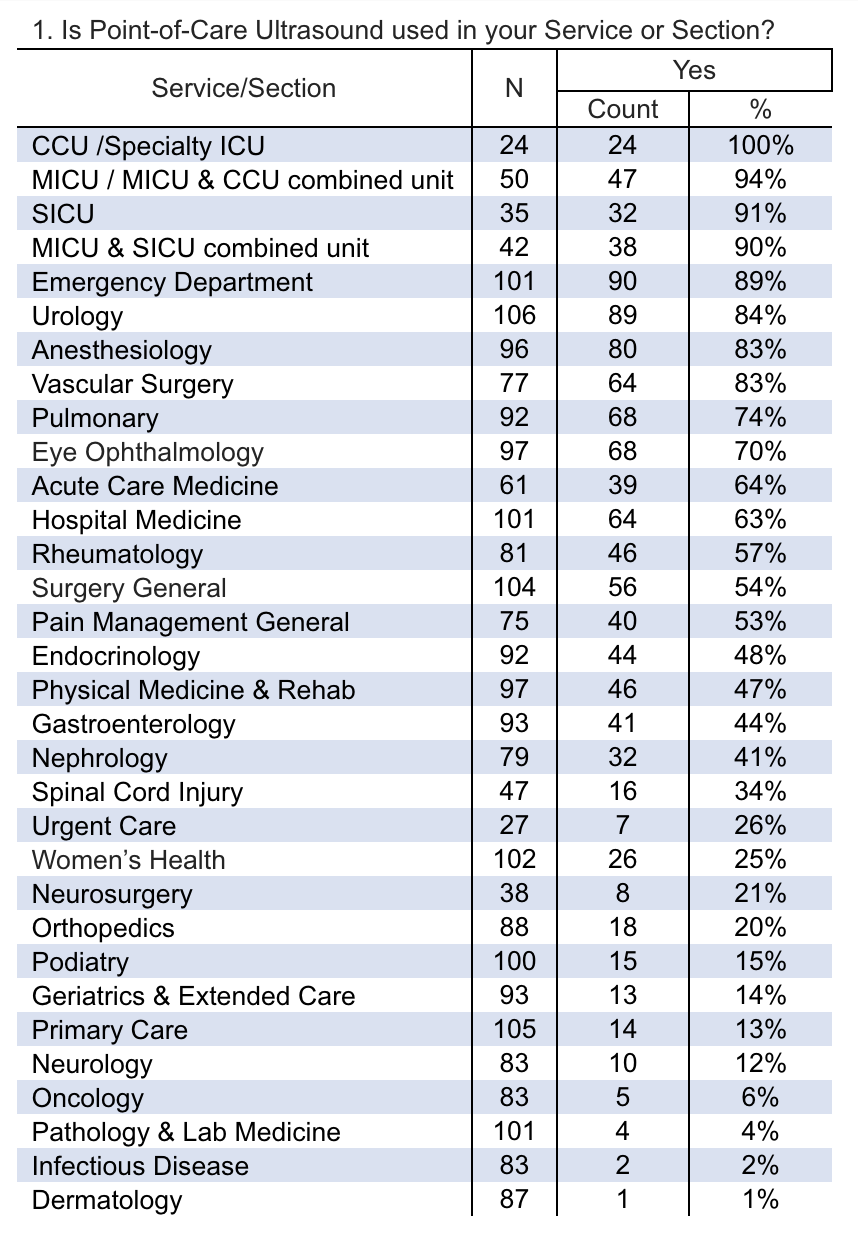


1b. Select the diagnostic and procedural POCUS applications routinely used by your Service or Section and then estimate the percentage of physicians that currently use POCUS for the diagnostic and procedural applications chosen.

***** All responses <5% were removed. In some instances, this resulted in no POCUS use for a specialty. A descriptor of higher use exams was added in these cases.**

| Diagnostic or Procedural Application | Count | % |
| --- | --- | --- |
| **All ICUs N=151** | | |
| Central Line Placement | 123 | 81% |
| Pleural Effusion | 108 | 72% |
| Arterial Line Placement | 106 | 70% |
| Volume Status (Inferior Vena Cava (IVC)/Internal Jugular (IJ)) | 105 | 70% |
| Left Ventricular Function | 100 | 66% |
| Thoracentesis | 99 | 66% |
| Pericardial Effusion | 92 | 61% |
| Pneumothorax | 85 | 56% |
| Paracentesis | 81 | 54% |
| Peripheral IV Access | 80 | 53% |
| Chest Tube | 70 | 46% |
| Peritoneal Fluid | 64 | 42% |
| Pulmonary Edema | 64 | 42% |
| Bladder | 52 | 34% |
| Urinary Retention | 51 | 34% |
| Advanced Hemodynamic Measurements (e.g., cardiac output, stroke volume) | 48 | 32% |
| Deep Vein Thrombosis (DVT) | 48 | 32% |
| Pneumonia | 42 | 28% |
| Hydronephrosis | 35 | 23% |
| Abscess | 33 | 22% |
| Peripherally Inserted Central Catheter (PICC) Placement | 33 | 22% |
| Arterial Flow | 31 | 21% |
| Focused Assessment with Sonography for Trauma (FAST) | 32 | 21% |
| Pericardiocentesis | 30 | 20% |
| Venous Mapping | 27 | 18% |
| Abdominal Aortic Aneurism (AAA) | 22 | 15% |
| Abscess Drainage | 22 | 15% |
| Intravascular Ultrasound | 18 | 12% |
| Joint Effusion | 18 | 12% |
| Lumbar Puncture | 14 | 9% |
| Biliary | 13 | 9% |
| Arthrocentesis | 11 | 7% |
| Pneumoperitoneum | 11 | 7% |
| Neck Mass | 11 | 7% |
| Lymph Nodes | 10 | 7% |
| Cellulitis | 8 | 5% |
| Optic Nerve Sheath Diameter | 8 | 5% |
| Small Bowel Obstruction | 8 | 5% |
| Peripheral Nerve Blocks | 7 | 5% |
| Endotracheal Intubation | 7 | 5% |
| Thyroid Gland | 7 | 5% |

| **Emergency Department N=101** | | |
| --- | --- | --- |
| Central Line Placement | 78 | 77% |
| Peripheral IV Access | 69 | 68% |
| Focused Assessment with Sonography for Trauma (FAST) | 63 | 62% |
| Urinary Retention | 55 | 54% |
| Abscess | 53 | 52% |
| Abdominal Aortic Aneurism (AAA) | 50 | 50% |
| Volume Status (Inferior Vena Cava (IVC)/Internal Jugular (IJ)) | 46 | 46% |
| Paracentesis | 45 | 45% |
| Pericardial Effusion | 44 | 44% |
| Peritoneal Fluid | 43 | 43% |
| Bladder | 42 | 42% |
| Pleural Effusion | 39 | 39% |
| Abscess Drainage | 38 | 38% |
| Pneumothorax | 35 | 35% |
| Hydronephrosis | 34 | 34% |
| Intrauterine Pregnancy | 32 | 32% |
| Biliary | 31 | 31% |
| Deep Vein Thrombosis (DVT) | 31 | 31% |
| Eye – Posterior Chamber (i.e., retinal detachment, vitreous detachment, etc.) | 30 | 30% |
| Left Ventricular Function | 26 | 26% |
| Thoracentesis | 26 | 26% |
| Foreign Body | 26 | 26% |
| Joint Effusion | 26 | 26% |
| Pulmonary Edema | 24 | 24% |
| Cellulitis | 24 | 24% |
| Foreign Body Removal | 21 | 21% |
| Arthrocentesis | 21 | 21% |
| Arterial Line Placement | 16 | 16% |
| Nephrolithiasis | 15 | 15% |
| Joint Injection | 15 | 15% |
| Chest Tube | 14 | 14% |
| Lumbar Puncture | 14 | 14% |
| Venous Mapping | 13 | 13% |
| Pericardiocentesis | 12 | 12% |
| Pneumonia | 12 | 12% |
| Uterus | 12 | 12% |
| Arterial Flow | 10 | 10% |
| Fractures | 10 | 10% |
| Ovaries | 9 | 9% |
| Peripherally Inserted Central Catheter (PICC) Placement | 8 | 8% |
| Optic Nerve Sheath Diameter | 8 | 8% |
| Bursitis | 8 | 8% |
| Intravascular Ultrasound | 7 | 7% |
| Peripheral Nerve Blocks | 7 | 7% |
| Appendicitis | 6 | 6% |
| Tendinopathies | 6 | 6% |
| Endotracheal Intubation | 5 | 5% |
| Small Bowel Obstruction | 5 | 5% |
| Pneumoperitoneum | 5 | 5% |
| Suprapubic Catheter | 5 | 5% |
|  |  |  |
| **Urgent Care N=27 (15% peritoneal fluid, 11%= cardiac, FAST, lung, AAA, PIV, soft tissue)** | | |

| **Hospital Medicine N=101** | | |
| --- | --- | --- |
| Paracentesis | 55 | 54% |
| Thoracentesis | 46 | 46% |
| Pleural Effusion | 45 | 45% |
| Central Line Placement | 42 | 42% |
| Peritoneal Fluid | 37 | 37% |
| Volume Status (Inferior Vena Cava (IVC)/Internal Jugular (IJ)) | 34 | 34% |
| Left Ventricular Function | 29 | 29% |
| Pericardial Effusion | 23 | 23% |
| Urinary Retention | 20 | 20% |
| Peripheral IV Access | 20 | 20% |
| Pulmonary Edema | 19 | 19% |
| Arterial Line Placement | 19 | 19% |
| Bladder | 16 | 16% |
| Pneumothorax | 14 | 14% |
| Pneumonia | 14 | 14% |
| Abscess | 14 | 14% |
| Deep Vein Thrombosis (DVT) | 13 | 13% |
| Hydronephrosis | 12 | 12% |
| Joint Effusion | 11 | 11% |
| Arthrocentesis | 10 | 10% |
| Abscess Drainage | 9 | 9% |
| Peripherally Inserted Central Catheter (PICC) Placement | 8 | 8% |
| Lumbar Puncture | 8 | 8% |
| Chest Tube | 7 | 7% |
| Biliary | 6 | 6% |
| Cellulitis | 6 | 6% |
| Advanced Hemodynamic Measurements (e.g., cardiac output, stroke volume) | 5 | 5% |
| Focused Assessment with Sonography for Trauma (FAST) | 5 | 5% |
| Abdominal Aortic Aneurism (AAA) | 5 | 5% |
| Joint Injection | 5 | 5% |
| **Urgent Care N=27 (15% peritoneal fluid, 11%= cardiac, FAST, lung, AAA, PIV, soft tissue)** | | |
| **Acute Care Medicine N=61** | | |
| Pleural Effusion | 32 | 52% |
| Paracentesis | 32 | 52% |
| Central Line Placement | 27 | 44% |
| Thoracentesis | 26 | 43% |
| Left Ventricular Function | 20 | 33% |
| Peritoneal Fluid | 20 | 33% |
| Volume Status (Inferior Vena Cava (IVC)/Internal Jugular (IJ)) | 19 | 31% |
| Pericardial Effusion | 18 | 30% |
| Peripheral IV Access | 18 | 30% |
| Pneumothorax | 17 | 28% |
| Urinary Retention | 17 | 28% |
| Pulmonary Edema | 14 | 23% |
| Bladder | 14 | 23% |
| Chest Tube | 13 | 21% |
| Arterial Line Placement | 12 | 20% |
| Peripherally Inserted Central Catheter (PICC) Placement | 11 | 18% |
| Pneumonia | 10 | 16% |
| Deep Vein Thrombosis (DVT) | 10 | 16% |
| Hydronephrosis | 9 | 15% |
| Arthrocentesis | 9 | 15% |
| Joint Injection | 8 | 13% |
| Thyroid Gland | 8 | 13% |
| Thyroid Biopsy | 7 | 11% |
| Pericardiocentesis | 6 | 10% |
| Abscess | 6 | 10% |
| Joint Effusion | 6 | 10% |
| Bursa Injection | 6 | 10% |
| Lumbar Puncture | 6 | 10% |
| Synovitis | 5 | 8% |
| Abscess Drainage | 5 | 8% |
| Venous Mapping | 4 | 7% |
| Endotracheal Intubation | 4 | 7% |
| Abdominal Aortic Aneurism (AAA) | 4 | 7% |
| Bursitis | 4 | 7% |
| Neck Mass | 4 | 7% |
| Advanced Hemodynamic Measurements (e.g., cardiac output, stroke volume) | 3 | 5% |
| Focused Assessment with Sonography for Trauma (FAST) | 3 | 5% |
| Tendinopathies | 3 | 5% |
| Cellulitis | 3 | 5% |
| Tendon Injection | 3 | 5% |
| Parathyroid Glands | 3 | 5% |

| **Anesthesiology N=96** | | |
| --- | --- | --- |
| Central Line Placement | 69 | 72% |
| Arterial Line Placement | 67 | 70% |
| Peripheral IV Access | 66 | 69% |
| Peripheral Nerve Blocks | 63 | 66% |
| Left Ventricular Function | 30 | 31% |
| Volume Status (Inferior Vena Cava (IVC)/Internal Jugular (IJ)) | 29 | 30% |
| Advanced Hemodynamic Measurements (e.g., cardiac output, stroke volume) | 28 | 29% |
| Pericardial Effusion | 26 | 27% |
| Pneumothorax | 24 | 25% |
| Pleural Effusion | 23 | 24% |
| Arterial Flow | 22 | 23% |
| Peripherally Inserted Central Catheter (PICC) Placement | 13 | 14% |
| Intravascular Ultrasound | 11 | 11% |
| Thoracentesis | 10 | 10% |
| Urinary Retention | 10 | 10% |
| Lumbar Puncture | 10 | 10% |
| Pulmonary Edema | 9 | 9% |
| Joint Injection | 9 | 9% |
| Venous Mapping | 8 | 8% |
| Pneumonia | 8 | 8% |
| Focused Assessment with Sonography for Trauma (FAST) | 8 | 8% |
| Deep Vein Thrombosis (DVT) | 7 | 7% |
| Abdominal Aortic Aneurism (AAA) | 6 | 6% |
| Pericardiocentesis | 5 | 5% |
| Endotracheal Intubation | 5 | 5% |
| Peritoneal Fluid | 5 | 5% |
| Joint Effusion | 5 | 5% |
| Bursa Injection | 5 | 5% |
| **Dermatology N=87 (“FB, bursa injection, other” all at 1%)** | | |
| **Endocrinology N=92** | | |
| Thyroid Gland | 41 | 45% |
| Thyroid Biopsy | 29 | 32% |
| Parathyroid Glands | 19 | 21% |
| Neck Mass | 16 | 17% |
| Lymph Node Biopsy | 5 | 5% |
| **Eye/Ophthalmology N=97** | | |
| Eye – Posterior Chamber (i.e., retinal detachment, vitreous detachment, etc.) | 64 | 66% |
| Optic Nerve Sheath Diameter | 11 | 11% |
| Other procedural applications used, please specify | 7 | 7% |

| \| Diagnostic or Procedural Application \| Count \| % \| \| --- \| --- \| --- \|     **Gastroenterology N=93** | | |
| --- | --- | --- | --- | --- | --- |
| Paracentesis | 21 | 23% |
| Peritoneal Fluid | 18 | 19% |
| Liver Biopsy | 12 | 13% |
| Biliary | 6 | 6% |
| Other procedural applications used, please specify | 5 | 5% |
| **Geriatrics & Extended Care N=93** | | |
| Urinary Retention | 9 | 10% |
| Bladder | 5 | 5% |
| **Infectious Disease N=83** *(One site uses for 17 applications = 1% use)* | | |
| **Nephrology N=79** | | |
| Central Line Placement | 20 | 25% |
| Urinary Retention | 18 | 23% |
| Hydronephrosis | 14 | 18% |
| Volume Status (Inferior Vena Cava (IVC)/Internal Jugular (IJ)) | 12 | 15% |
| Bladder | 11 | 14% |
| Nephrolithiasis | 7 | 9% |
| Other procedural applications used, please specify | 7 | 9% |
| Pericardial Effusion | 6 | 8% |
| Pulmonary Edema | 6 | 8% |
| Pleural Effusion | 5 | 6% |
| Left Ventricular Function | 4 | 5% |
| Peritoneal Fluid | 4 | 5% |
| Paracentesis | 4 | 5% |
| **Neurology N=83 (other 4%, peripheral nerve blocks, tendinopathies, Joint effusion 2%)** | | |
| **Neurosurgery N=38** | | |
| Other procedural applications used, please specify | 7 | 18% |
| **Oncology N=83** | | |
| Pleural Effusion | 4 | 5% |

1b. (continued)

| Diagnostic or Procedural Application | Count | % |
| --- | --- | --- |
| **Oncology N=83 (vascular access and biopsy 1%)** | | |

| **Orthopedics N=88** | | |
| --- | --- | --- |
| Joint Injection | 15 | 17% |
| Shoulder/Rotator Cuff | 10 | 11% |
| Bursa Injection | 10 | 11% |
| Tendon Injection | 10 | 11% |
| Joint Effusion | 8 | 9% |
| Tendinopathies | 6 | 7% |
| Arthrocentesis | 6 | 7% |
| Abscess | 5 | 6% |
| Bursitis | 5 | 6% |
| Foreign Body | 4 | 5% |
| **Pain Management – General N=75** | | |
| Joint Injection | 30 | 40% |
| Bursa Injection | 27 | 36% |
| Peripheral Nerve Blocks | 23 | 31% |
| Tendon Injection | 22 | 29% |
| Tendinopathies | 18 | 24% |
| Bursitis | 18 | 24% |
| Shoulder/Rotator Cuff | 17 | 23% |
| Joint Effusion | 15 | 20% |
| Arthrocentesis | 11 | 15% |
| Peripheral IV Access | 10 | 13% |
| Central Line Placement | 10 | 13% |
| Synovitis | 10 | 13% |
| Arterial Line Placement | 9 | 12% |
| Left Ventricular Function | 8 | 11% |
| Volume Status (Inferior Vena Cava (IVC)/Internal Jugular (IJ)) | 8 | 11% |
| Pneumothorax | 7 | 9% |
| Arterial Flow | 7 | 9% |
| Pericardial Effusion | 6 | 8% |
| Advanced Hemodynamic Measurements (e.g., cardiac output, stroke volume) | 6 | 8% |
| Pleural Effusion | 5 | 7% |
| Peripherally Inserted Central Catheter (PICC) Placement | 5 | 7% |
| Pulmonary Edema | 4 | 5% |
| Deep Vein Thrombosis (DVT) | 4 | 5% |
| Intravascular Ultrasound | 4 | 5% |

| **Physical Medicine and Rehabilitation (PM&R) N=97** | | |
| --- | --- | --- |
| Joint Injection | 43 | 44% |
| Bursa Injection | 39 | 40% |
| Shoulder/Rotator Cuff | 38 | 39% |
| Tendon Injection | 35 | 36% |
| Tendinopathies | 34 | 35% |
| Bursitis | 33 | 34% |
| Joint Effusion | 32 | 33% |
| Synovitis | 26 | 27% |
| Arthrocentesis | 26 | 27% |
| Peripheral Nerve Blocks | 13 | 13% |
| Other procedural applications used, please specify | 9 | 9% |
| Foreign Body | 5 | 5% |
| **Pathology & Laboratory Medicine N=101** (2% use Lymph node & thyroid biopsies, neck mass) | | |
| **Podiatry N=100** | | |
| Tendinopathies | 11 | 11% |
| Joint Injection | 11 | 11% |
| Foreign Body | 10 | 10% |
| Bursa Injection | 10 | 10% |
| Tendon Injection | 10 | 10% |
| Arthrocentesis | 10 | 10% |
| Joint Effusion | 9 | 9% |
| Abscess | 8 | 8% |
| Bursitis | 7 | 7% |
| Foreign Body Removal | 7 | 7% |
| Fractures | 6 | 6% |
| Cellulitis | 5 | 5% |
| Synovitis | 5 | 5% |
| **Primary Care N=105** | | |
| Bladder | 6 | 6% |
| Urinary Retention | 5 | 5% |

| **Pulmonary N=92** | | |
| --- | --- | --- |
| Pleural Effusion | 66 | 72% |
| Thoracentesis | 66 | 72% |
| Pneumothorax | 49 | 53% |
| Central Line Placement | 49 | 53% |
| Chest Tube | 45 | 49% |
| Volume Status (Inferior Vena Cava (IVC)/Internal Jugular (IJ)) | 42 | 46% |
| Arterial Line Placement | 41 | 45% |
| Left Ventricular Function | 37 | 40% |
| Pulmonary Edema | 35 | 38% |
| Pericardial Effusion | 30 | 33% |
| Paracentesis | 29 | 32% |
| Pneumonia | 26 | 28% |
| Peripheral IV Access | 26 | 28% |
| Deep Vein Thrombosis (DVT) | 22 | 24% |
| Peritoneal Fluid | 19 | 21% |
| Bladder | 16 | 17% |
| Urinary Retention | 14 | 15% |
| Hydronephrosis | 12 | 13% |
| Venous Mapping | 11 | 12% |
| Advanced Hemodynamic Measurements (e.g., cardiac output, stroke volume) | 11 | 12% |
| Focused Assessment with Sonography for Trauma (FAST) | 9 | 10% |
| Peripherally Inserted Central Catheter (PICC) Placement | 9 | 10% |
| Lymph Nodes | 8 | 9% |
| Lumbar Puncture | 8 | 9% |
| Arterial Flow | 7 | 8% |
| Lymph Node Biopsy | 7 | 8% |
| Abscess | 6 | 7% |
| **Rheumatology N=81** | | |
| Synovitis | 43 | 53% |
| Joint Effusion | 42 | 52% |
| Joint Injection | 36 | 44% |
| Arthrocentesis | 36 | 44% |
| Tendinopathies | 35 | 43% |
| Bursa Injection | 34 | 42% |
| Bursitis | 33 | 41% |
| Tendon Injection | 29 | 36% |
| Shoulder/Rotator Cuff | 28 | 35% |
| Cellulitis | 5 | 6% |

| Diagnostic or Procedural Application | Count | % |
| --- | --- | --- |
| **Surgery (General) N=104** | | |
| Central Line Placement | 39 | 38% |
| Arterial Line Placement | 21 | 20% |
| Peripheral IV Access | 19 | 18% |
| Abscess | 18 | 17% |
| Abscess Drainage | 16 | 15% |
| Thoracentesis | 15 | 14% |
| Peritoneal Fluid | 15 | 14% |
| Pleural Effusion | 14 | 13% |
| Thyroid Gland | 14 | 13% |
| Arterial Flow | 13 | 13% |
| Paracentesis | 12 | 12% |
| Urinary Retention | 12 | 12% |
| Prostate Biopsy | 12 | 12% |
| Thyroid Biopsy | 12 | 12% |
| Venous Mapping | 11 | 11% |
| Prostate | 11 | 11% |
| Peripherally Inserted Central Catheter (PICC) Placement | 11 | 11% |
| Bladder | 10 | 10% |
| Parathyroid Glands | 10 | 10% |
| Neck Mass | 10 | 10% |
| Volume Status (Inferior Vena Cava (IVC)/Internal Jugular (IJ)) | 9 | 9% |
| Biliary | 9 | 9% |
| Focused Assessment with Sonography for Trauma (FAST) | 9 | 9% |
| Deep Vein Thrombosis (DVT) | 9 | 9% |
| Pericardial Effusion | 8 | 8% |
| Suprapubic Catheter | 8 | 8% |
| Foreign Body | 8 | 8% |
| Lymph Node Biopsy | 8 | 8% |
| Chest Tube | 7 | 7% |
| Liver Biopsy | 7 | 7% |
| Intravascular Ultrasound | 7 | 7% |
| Joint Effusion | 7 | 7% |
| Foreign Body Removal | 7 | 7% |
| Abdominal Aortic Aneurism (AAA) | 6 | 6% |
| Lymph Nodes | 6 | 6% |
| Breast Biopsy | 6 | 6% |
| Left Ventricular Function | 5 | 5% |
| Pneumothorax | 5 | 5% |
| Uterus | 5 | 5% |
| Peripheral Nerve Blocks | 5 | 5% |
| Cellulitis | 5 | 5% |
| Bursitis | 5 | 5% |
|  | Count | % |
| **Spinal Cord Injury N=47** | | |
| Joint Injection | 10 | 21% |
| Bursitis | 9 | 19% |
| Bursa Injection | 9 | 19% |
| Shoulder/Rotator Cuff | 8 | 17% |
| Joint Effusion | 8 | 17% |
| Tendon Injection | 8 | 17% |
| Tendinopathies | 7 | 15% |
| Synovitis | 7 | 15% |
| Arthrocentesis | 6 | 13% |
| Other procedural applications used, please specify | 6 | 13% |
| Bladder | 5 | 11% |
| Urinary Retention | 4 | 9% |
| **Urology N=106** | | |
| Prostate Biopsy | 81 | 76% |
| Prostate | 66 | 62% |
| Urinary Retention | 64 | 60% |
| Bladder | 56 | 53% |
| Suprapubic Catheter | 33 | 31% |
| Hydronephrosis | 16 | 15% |
| Nephrolithiasis | 8 | 8% |
| Nephrostomy Tube | 6 | 6% |
| **Vascular Surgery N=77** | | |
| Arterial Flow | 53 | 69% |
| Central Line Placement | 45 | 58% |
| Arterial Line Placement | 40 | 52% |
| Intravascular Ultrasound | 40 | 52% |
| Venous Mapping | 37 | 48% |
| Deep Vein Thrombosis (DVT) | 27 | 35% |
| Abdominal Aortic Aneurism (AAA) | 26 | 34% |
| Peripheral IV Access | 24 | 31% |
| Peripherally Inserted Central Catheter (PICC) Placement | 17 | 22% |
| Other procedural applications used, please specify | 12 | 16% |
| Abscess | 5 | 6% |
